# A Gamified N-back App for Identifying Mild-cognitive Impairment in Older Adults

**DOI:** 10.31662/jmaj.2024-0217

**Published:** 2024-12-20

**Authors:** Naohiro Murata, Shozo Nishii, Ryoya Usuha, Asuka Kodaka, Masako Fujimori, Haruka Sugawara, Takashi Kiriyama, Hirotake Uchikado, Yasuo Okumura, Takanori Takebe

**Affiliations:** 1 Communication Design Center, Advanced Medical Research Center, Yokohama City University Graduate School of Medicine, Yokohama, Japan; 2 Chibune General Hospital, Social Medical Corporation Aijinkai, Osaka, Japan; 3 Tokyo University of the Arts, Art Media Center, Tokyo, Japan; 4 Tokyo University of the Arts, Graduate School of Film and New Media, Yokohama, Japan; 5 Memory Care Clinic Shonan, Hiratsuka, Japan; 6 Division of Cardiology, Department of Medicine, Nihon University School of Medicine, Tokyo, Japan; 7 Department of Genome Biology, Graduate School of Medicine, and WPI Premium Research Institute for Human Metaverse Medicine (WPI-PRIMe), Osaka University, Osaka, Japan; 8 The Institute of Science Tokyo, Tokyo, Japan; 9 Division of Gastroenterology, Hepatology & Nutrition and Developmental Biology, Cincinnati Children’s Hospital Medical Center, Cincinnati, USA; 10 Department of Pediatrics, University of Cincinnati College of Medicine, Cincinnati, USA; 11 Center for Stem Cell and Organoid Medicine (CuSTOM), Cincinnati Children’s Hospital Medical Center, Cincinnati, USA

**Keywords:** Digital Health, Dementia, Mild-cognitive impairment, Diagnostic screening, N-back task, Gamification, Working memory, Happiness-driven

## Abstract

**Introduction:**

Despite a dramatic increase in the incidence of mild-cognitive impairment (MCI) and early dementia, accessible and engaging screening methods for older adults are lacking. Gamification has gained attention in the self-management of various health conditions, making it a promising avenue for dementia screening. This study aimed to evaluate a gamified mobile application for the early detection of cognitive impairment associated with dementia.

**Methods:**

The gamified app and the Mini-Mental State Examination (MMSE) were administered to 138 participants. The game, based on the N-back working memory task, simulates a restaurant scenario where players cook curries with hidden ingredients to fulfill customer orders, with the difficulty increasing in each round. The correlations between MMSE scores and game metrics were analyzed, and the game metrics were compared between the normal and impaired groups.

**Results:**

Among the 138 older adult participants, the game metrics such as level reached, accuracy, response times, tap times, and swipe times exhibited significant correlations with scores on the MMSE, a standard cognitive screening tool (r = 0.42, 0.419, −0.575, −0.484, and −0.667, respectively; *P* < 0.05 for all). The participants were divided into the normal (≥28) and impaired (<28) groups based on the MMSE cutoff values. The impaired group had significantly worse performance on all game metrics. After multivariate adjustment, average swipe time emerged as the strongest predictor, achieving 70.8% sensitivity and 80.6% specificity in detecting impairment using a 3.31-s cutoff (area under the curve = 0.820).

**Conclusions:**

This classification accuracy was comparable to standard dementia screening tests. These results indicate the potential use of gamification with joyous experience for older adults to enable scalable cognitive screening beyond conventional testing paradigms.

Dementia is a growing global health crisis; it is estimated to affect approximately 75 million individuals by 2030, escalating to 132 million by 2050 ^(1)^. Despite its increasing prevalence, low awareness and stigma surrounding the condition often impede timely diagnosis and care ^(2)^. By the time cognitive impairment becomes noticeable to patients, their close relatives, or physicians, the cascade of events leading to full-blown Alzheimer’s disease (AD) may have become irreversible without disease-modifying therapy ^(3)^. Thus, early detection and proactive intervention for dementia have become urgent priorities.

Early therapeutic intervention has proven effective in delaying symptom progression ^(3), (4)^. For example, antiamyloid beta antibody drugs have shown promise in reducing amyloid markers and mitigating cognitive decline in AD ^(5)^ . The current critical challenges involve the deployment of reliable means to detect the early signs of cognitive impairments during dementia care. While simple cognitive tests exist, they are often perceived as demeaning, potentially decreasing self-esteem and adherence. In fact, 70% of individuals who have undergone dementia screening tests reported feeling distressed ^(6)^. This lack of accessible and acceptable early screening methods perpetuates delayed diagnosis and treatment for most individuals with mild-cognitive impairment (MCI) or early dementia.

The very early stages of AD are marked by executive dysfunction and working memory (WM) impairments, in addition to episodic memory deficits. These cognitive deficits, which begin during the MCI phase, may indicate progression to AD. Consequently, there is a need for studies that can detect and monitor changes in WM, attention, and executive function in individuals with MCI and older individuals with no impairment ^(7)^. The N-back task is a cognitive task where participants are presented with numbers or letters at regular intervals and must respond when the current number or letter matches the one presented N items ago. The N-back task is a widely used WM task in large, cross-sectional studies involving younger, middle-aged, and older adults ^(8)^. It has shown efficacy in differentiating MCI from AD via brainwave patterns ^(9)^. Responses to the WM task could not only improve the early diagnosis of MCI and AD but also be envisioned in assessing the potential progression from MCI to AD, after further verification through longitudinal studies.

Gamification has gained attention in the self-management of various health conditions, making it a promising avenue for dementia screening ^(10)^. Mobile applications present a promising solution for enhancing screening and patient management in dementia care ^(5)^. While existing dementia screening apps such as MoCA and BrainTest^Ⓡ^ have demonstrated validity ^(11), (12), (13)^, many other apps, such as eSLUMS, are designed for professional administration and often require specialist interpretation and clinical visits ^(11)^. This approach fails to resolve stigma and accessibility barriers ^(11)^. Therefore, the development of simplified and engaging screening tests on mobile devices is crucial for the wide adoption of digital medicine in dementia care, potentially reducing psychological burden and improving accessibility.

By creating an engaging and less intimidating experience, we hypothesized that gamified screening tools may help overcome the distress often associated with traditional cognitive tests, potentially increasing adherence and early detection rates. Furthermore, a decline in dexterity for operating smart devices may reflect a decrease in cognitive function ^(14)^, allowing mobile apps to collect data not only on cognitive tasks but also on usability. To test this, we developed a gamified mobile application based on the N-back task, focusing on WM. By combining the benefits of mobile technology, gamification ^(15), (16)^, and evidence-based cognitive assessment, this approach will provide a more user-friendly and effective method for the early detection of cognitive impairment, potentially leading to earlier interventions and better outcomes for individuals at risk of dementia.

## Methods

### Participants

**Figure 1.**
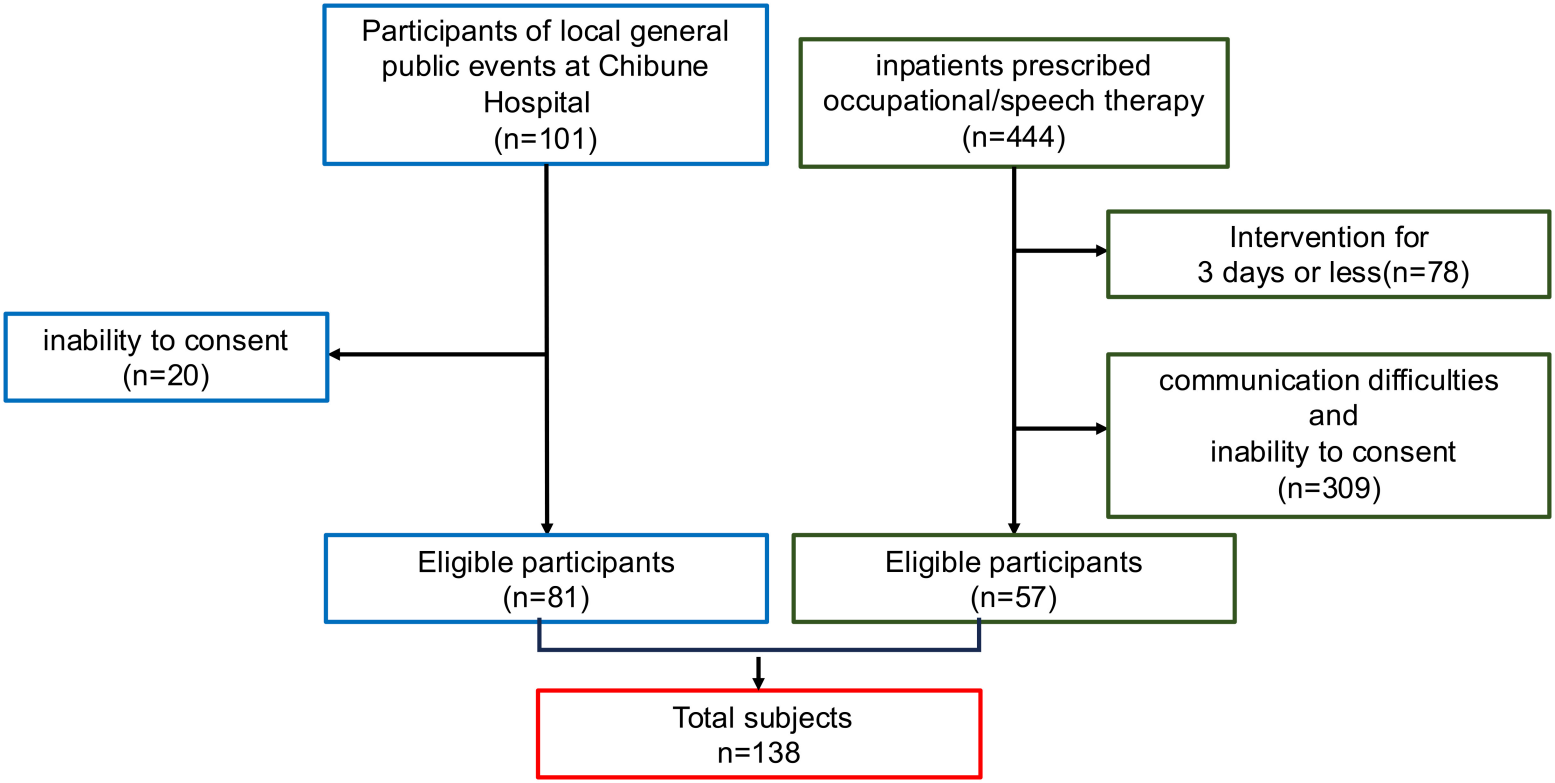
Flow chart for participant enrollment.

A total of 138 participants (43 men and 95 women, mean age 75.4 ± 10.3 years) were recruited from local general public events at Chibune Hospital (September 2022-October 2023) (n = 81) and inpatients prescribed occupational/speech therapy (October-December 2022) (n = 57) (Figure 1). Patients with communication difficulties and inability to consent were excluded. Potential gender distribution bias exists due to voluntary recruitment (Table 1).[Table table2]

**Table 1.** Participant Characteristics and Game Metrics between the Normal and Cognitive Impairment Groups.

		Total (n = 138)	Normal (n = 72)	Cognitive impairment (n = 66)	*P*-value
Participant characteristics	Age (year)	75.8 ± 10.8	71.3 ± 11.5	80.7 ± 7.6	<0.001
	Male	43 (31%)	15 (21%)	28 (42%)	0.009
	MMSE	26.3 ± 4.0	29.2 ± 1.2	23.2 ± 3.62	<0.001
Game metrics	Level reached	4.74 ± 1.13	5.18 ± 1.14	4.32 ± 1.00	<0.001
	Accuracy (%)	68.5 ± 11.7	72.1 ± 10.7	65.1 ± 11.9	<0.001
	Response time (s)	6.25 ± 4.04	4.60 ± 1.78	8.21 ± 4.56	<0.001
	Tap time (s)	3.51 ± 2.21	3.21 ± 1.33	4.81 ± 2.22	<0.001
	Swipe time (s)	3.85 ± 1.90	2.53 ± 1.11	4.94 ± 3.29	<0.001

a. Summary of key participant demographics: age, sex, recruitment details. b. List of game metrics recorded: accuracy, level, response times, tap times, swipe times. Values are expressed as mean ± standard deviation. c. The cognitive impairment group (MMSE ≤ 27) performed significantly worse than the normal group across all game metrics, including accuracy, level, response times, tap times, and swipe times.

**Table 2. table2:** Correlations with MMSE.

	Correlation coefficient	*P*-value
Level reached	0.420	<0.001
Accuracy (%)	0.419	<0.001
Response time (sec)	−0.575	<0.001
Tap time (sec)	−0.484	<0.001
Swipe time (sec)	−0.667	<0.001

Accuracy and level reached showed positive correlations with MMSE scores (r = 0.419 and r = 0.42, *P* < 0.05). Conversely, response time, tap time, and swipe time were negatively correlated (r = −0.575, −0.484, and −0.667, respectively, *P* < 0.05).

### Game description

The gamified app simulates a restaurant where players take orders by preparing curry dishes with hidden ingredients matching customer preferences. It consists of two phases:

1. Memory Phase: Players cook curry, adding and memorizing the placement of hidden ingredients.

2. Answer Phase: Players select curries with the requested hidden ingredients to serve customers.

The game uses the N-back task, gradually increasing difficulty by requiring players to memorize N additional ingredients after correctly fulfilling N orders. In-game revenue reflects performance based on correct orders.

### Objective measures

Accuracy (the percentage of correctly provided ordered items), level reached (number of items remembered at once), response time (average time to fulfill an order after receiving it), tap time (average time to tap the screen to cut one ingredient), and swipe time (average time to swipe the screen to put one ingredient into the pot) during the Memory Phase were recorded as objective performance measures.

### Gameplay duration

**Figure 2.**
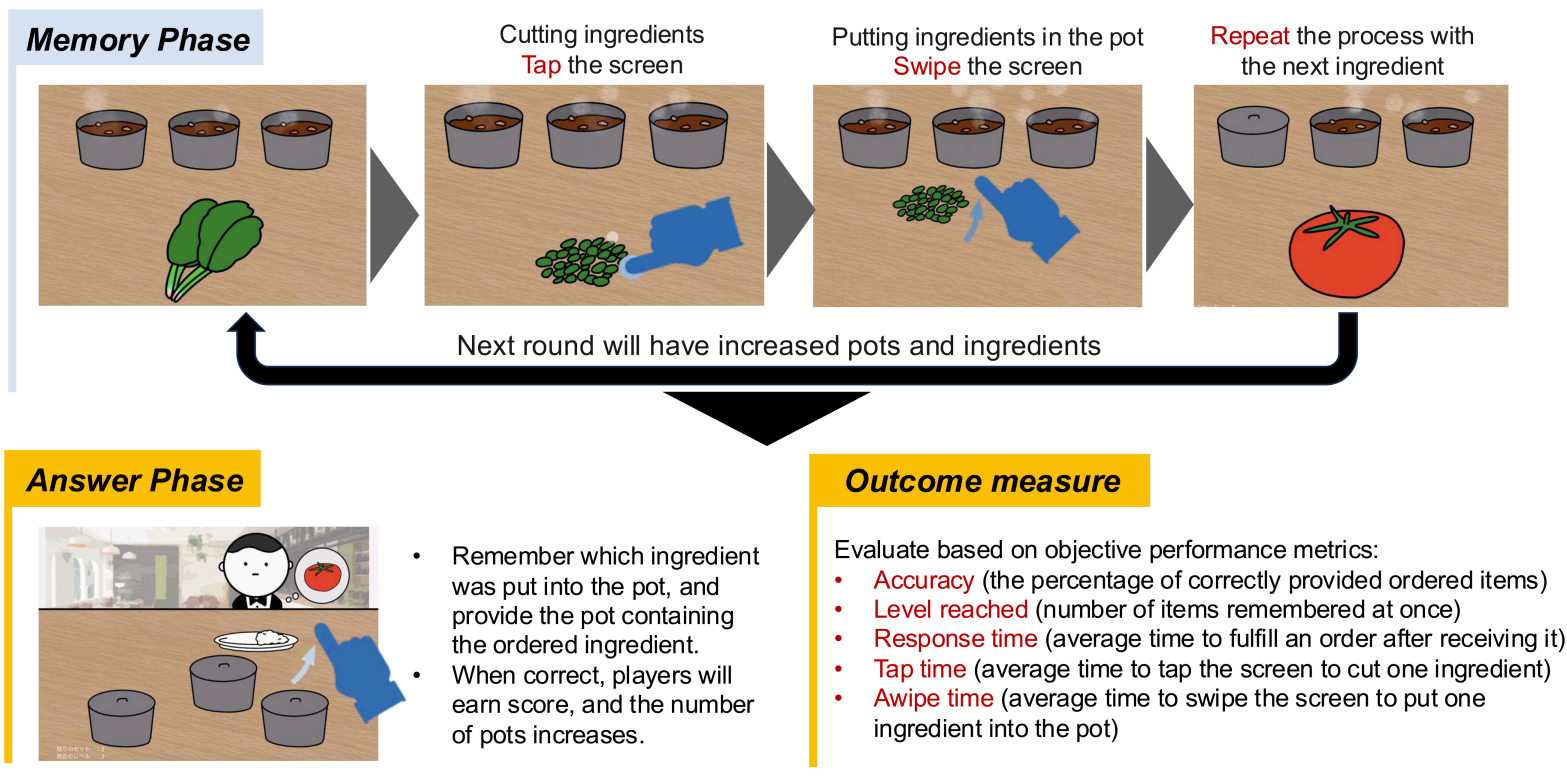
Gamification design for the N-back task The gamified app simulates a restaurant scenario with two main phases: a. Memory Phase: Players roleplay to cook curry, add hidden ingredients for flavor, and memorize their placements. b. Answer Phase: Players select and serve curries containing the ingredients requested by each customer.

Each solo gameplay session lasted approximately 5-10 min. After watching a demo video on how to play the game, the participants proceeded to play it themselves. Figure 2 presents the gameplay process.

### Equipment used

The participants played on third-generation iPad Airs.

### Evaluation

The participants completed the game and the Mini-Mental State Examination (MMSE) ^(17)^, the standard AD screening test ^(18)^. Correlations between the MMSE scores and game metrics were examined. Based on literature ^(19), (20)^, the participants were divided into the normal (MMSE ≥ 28) and cognitive impairment (MMSE ≤ 27) groups. Group differences in the game metrics were assessed.

### Statistics

The sample size was determined to be between 80 and 100 cases based on previous similar studies ^(12), (13), (21)^. For this study, it was set to over 100 cases. Continuous variables were expressed as mean ± standard deviation, whereas nominal variables were expressed as count and percentages. Spearman’s correlation was employed to examine the associations between the game metrics and the MMSE scores. Unpaired *t*-tests or Mann-Whitney’s *U* tests were used to compare the game metrics between the groups after assessing normality. Multivariate logistic regression was used to identify age and sex, and the game metrics. Furthermore, the variance inflation factor (VIF) was calculated to evaluate the presence of multicollinearity. A VIF value exceeding 2.0 was considered to indicate high multicollinearity and excluded the explanatory variables in the multivariate logistic regression analysis. Receiver operating characteristic (ROC) curves were used to determine the cutoff values for significant factors. Statistical analysis was conducted using the EZR software. ver 1.60 (Easy R, Saitama Medical Center, Jichi Medical University, Japan, Saitama) ^(22)^ The significance level was set at 5% for all analyses.

### Ethics

This study was approved by the Chibune Hospital Ethics Committee in July 2022 (No. 20230315A). All participants provided written informed consent.

## Results

### Participant characteristics

A total of 138 older adults participated in the study. Their demographic information is summarized in Table 1. The mean age of the participants was 75.8 years (SD = 10.8), indicating a sample representative of the elderly population. There were 43 men (31%) and 95 women (69%), indicating a notably skewed gender distribution. The sample size was sufficient to conduct meaningful analyses and draw preliminary conclusions regarding the efficacy of the gamified screening tool.

### Correlation between MMSE and the game metrics

A moderate positive correlation was observed between the MMSE scores and game accuracy (r = 0.419, *P* < 0.05) as well as the level reached in the game (r = 0.42, *P* < 0.05).

Significant negative correlations were observed between the MMSE scores and various operation times. The average tap time (r = −0.484, *P* < 0.05), swipe time (r = −0.667, *P* < 0.05), and response time (r = −0.575, *P* < 0.05) showed inverse relationships with the MMSE scores.

### Comparison between the normal and cognitive impairment groups

To further investigate the discriminative ability of the game, the participants were stratified into two groups based on the established MMSE cutoff values: normal cognitive function (MMSE ≥ 28, n = 72) and cognitive impairment (MMSE ≤ 27, n = 66). This classification aligns with widely accepted standards in cognitive assessment literatures ^(19), (20)^, allowing for meaningful group comparisons. The detailed participant characteristics and game metrics for each group are presented in Table 1.

Significant differences were observed between the two groups across all game metrics. The cognitively impaired group achieved lower levels, demonstrated reduced accuracy, exhibited slower response times, and showed slower tap and swipe times.

### Predictive performances for cognitive impairment

To assess the predictive power of game metrics for cognitive impairment status, a multivariate logistic regression was performed. This analysis controlled for age and sex, which are crucial factors in cognitive assessment. To avoid multicollinearity, only tap time and swipe time (VIF < 2.0) were included in the model alongside demographic variables (Table 3).

**Table 3.** Predicting Cognitive Impairment.

Independent variable	OR	*P*-value	95% CI	VIF
Age	1.09	0.006	1.02-1.15	1.32
Sex	3.92	0.008	1.41-10.9	1.19
Tap time	1.25	0.22	0.88-1.78	1.28
Swipe time	1.58	0.02	1.07-2.32	1.37
Level reached				2.36
Accuracy				2.37
Response time				2.14

a. Variables with VIF values exceeding 2.0 were considered as indicators of high multicollinearity and were excluded from the analysis. b. Logistic regression with cognitive impairment as the outcome and age and game metrics as predictors. c. Average swipe time emerged as the significant predictor of impairment status (*P* < 0.05).

Average swipe time emerged as a significant predictor of cognitive impairment status (*P* < 0.05).

**Figure 3.**
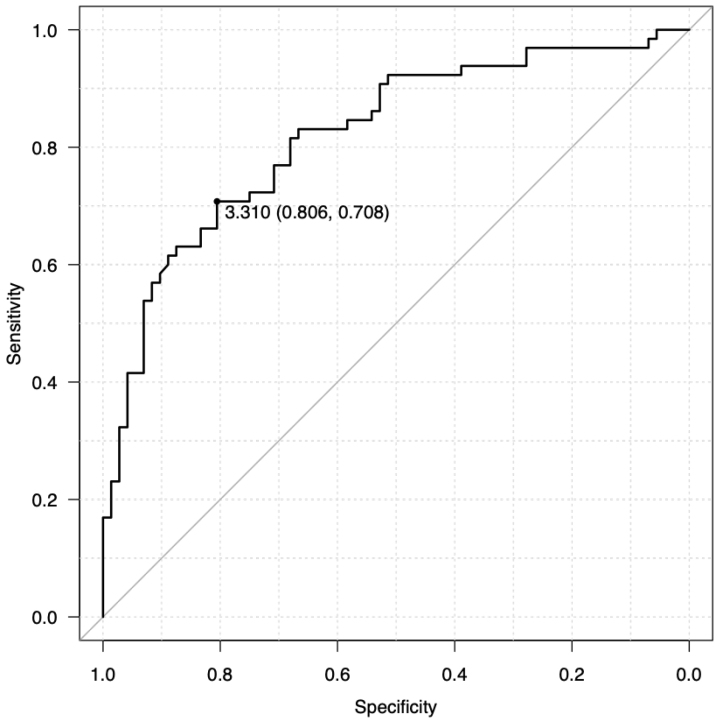
Receiver operating characteristic (ROC) curve analysis of swipe times in N-back games The ROC analysis identified average swipe time as a significant predictor of cognitive impairment status. The optimal cutoff value was 3.310 s, yielding 70.8% sensitivity and 80.6% specificity in detecting impairment (AUC = 0.820).

To further evaluate the discriminative ability of average swipe time, an ROC curve analysis was conducted. The area under the curve (AUC) of 0.820 indicates good discriminative performance (Figure 3).

A cutoff value of 3.310 s for average swipe time was identified as optimal, yielding a sensitivity of 70.8% and a specificity of 80.6%. These values indicate that the swipe time metric can correctly identify 70.8% of individuals with cognitive impairment (true positives) while also correctly classifying 80.6% of cognitively normal individuals (true negatives). This performance is comparable to or exceeds existing screening tools such as BrainTest^Ⓡ^^(13)^, eSLUMS ^(23)^, and finger-tapping tests (Table 4) ^(24)^.

**Table 4.** Screening Performance.

Inspection	Method	Sensitivity	Specificity	Time	Examiner
HDS-R	Paper	93%	86%	10 min	Health care workers
MMSE	Paper	45%-60%	65-90%	10 min	Health care workers
MoCA-J	Paper	100%	87%	10 min	Health care workers
BrainTest^Ⓡ^	Application	71%	90%	10-15min	The person or a caregiver
eSLUMS	Application	98%-100%	98%-100%	7-10min	Health care workers
Finger Tapping Test	Physical measurement	77%	67%	1-2min	Health care workers
This game	Physical measurement	70.8%	80.6%	10min	The person

With a swipe time cutoff of 3.31 s, this gamified assessment demonstrated screening accuracy comparable to that of existing tests, such as BrainTest^Ⓡ^ and eSLUMS.

## Discussion

Our findings indicate a clear association between cognitive function, as assessed by MMSE scores, and game performance. Participants with higher MMSE scores consistently achieved greater accuracy and advanced to more complex levels within the game, suggesting the potential of this gamified approach for use as a valid tool for cognitive assessment. Conversely, individuals with lower MMSE scores, indicative of reduced cognitive abilities, exhibited slower reaction times and delayed responses across all the measured tasks. These results reinforce the utility of the game-based platform in the differentiation of cognitive abilities and tracking of performance outcomes.

Tablet-based interventions have demonstrated feasibility for older adults, including those with dementia ^(25)^. However, conditions such as AD and MCI can impair motor functions, including fine motor skills and processing speed ^(26)^, which manifest as slower performance on tasks like zipper closure ^(27)^ and finger tapping ^(24)^. Significant negative correlations were observed between MMSE scores and rhythm and reaction times on smartphone devices ^(14)^, consistent with the findings of this study. This suggests that lower cognitive function, as measured by MMSE, corresponds to worse in-game performance. A decline in motor functions such as walking can even precede the onset of cognitive impairment by over a decade ^(28), (29)^. The significant correlations between game metrics involving motor responses (tapping, swiping, reaction time) and MMSE scores are consistent with our observation. The significant correlations across all game metrics provide initial evidence for the feasibility of using this gamified approach to screen for cognitive impairment. These consistent differences across multiple metrics strengthen the case for the ability of the game to differentiate between individuals with normal cognitive function and those with potential impairment. The multifaceted nature of the game, including aspects such as working memory, response speed, and operability like swiping, suggests that it captures a broad spectrum of functions that may be affected by cognitive decline. Similar to other studies ^(30), (31)^, age was significantly associated with cognitive impairment. However, even after adjusting for multiple variables, swipe time stood out as a strong, independent predictor of cognitive impairment. Sex differences were also observed. It is believed that fine motor skills decline with age more in men than in women ^(32)^ and that cognitive decline may further exacerbate this decrease in fine motor skills. Notably, a swipe time cutoff of 3.31 s demonstrated an impressive 70.8% sensitivity and 80.6% specificity, comparable to existing screening tools such as BrainTest^Ⓡ^^(13)^, eSLUMS ^(23)^, and finger-tapping tests ^(24)^. These results indicate that beyond correlations with overall cognitive status, certain game metrics, particularly processing speed as reflected in swipe time, can differentiate and predict the presence of cognitive deficits with promising accuracy. This provides support for using gamified assessments such as this app as an accessible screening tool for conditions like MCI.

As people age, they tend to prioritize choices that optimize their emotional state and psychological well-being ^(33)^. Research suggests that the amygdala of an aging brain shows decreased reactivity to negative information while sparing or even increasing its responsiveness to positive stimuli ^(34)^. In addition, older adults generally heighten their ability to avoid risks compared with younger individuals ^(35), (36)^. These age-related changes in cognitive and emotional processing suggest that the implementation of measures aimed at achieving psychological well-being is likely to promote healthy behaviors and potentially improve adherence to screening and early intervention programs. A key advantage of the gamified approach is increased engagement and independence compared with conventional screening tests ^(37)^ as gamification can reduce perceived burden and stigma around testing ^(38)^ while improving health-directed behaviors, such as physical activity ^(39)^. Contrary to existing apps such as BrainTest^Ⓡ^, which digitizes paper tests, this gamified approach validates a novel screening paradigm focused on behavioral metrics rather than conventional cognitive tasks. This will potentially expand accessibility beyond just health-conscious individuals to engage wider populations through an intuitive game format. There are cases where treatment adherence is improved through the use of games ^(40)^. Fostering positive emotions, such as “fun” or “want to try,” may be effective health promotion strategies compared with those relying on rational appeals to present the benefits of traditional health behaviors. Designing mechanisms for feedback and social interactions within games could further enhance real-world impact. While further refinements are needed, gamification shows promise for scalable, accessible cognitive screening aligned with self-management philosophies in digital health.

The limitations of this study include sex distribution bias in this sample due to the inclusion of data from a public event. This bias arises from the collection of samples at local events. Women’s opportunities for social participation remain relatively stable with aging, whereas men experience a sharp decline ^(41)^. In addition, people who are more socially engaged tend to have lower health risks ^(42)^, which may explain the higher participation of cognitively normal women. Furthermore, the lack of detailed medical history prevented the examination of potential confounding factors. Despite these limitations, multivariate analysis accounting for variables such as age and sex confirmed that swipe time is a strong independent predictor, supporting its potential in predicting cognitive impairment. Moreover, it is necessary to investigate past experience using smart devices. Reproducibility across devices is also needed given the focus on motor/operational factors over WM. Developing complementary games assessing memory alongside motor functions may optimize screening capabilities.

## Article Information

This article is based on the study, which received the Medical Research Encouragement Prize of The Japan Medical Association in 2023.

### Conflicts of Interest

None

### Sources of Funding

This work was supported by JST COI Grant Number JPMJPF2105, JSPS KAKENHI Grant Numbers JP20K02972, the grant for 2021-2023 Strategic Research Promotion (Nos. SK2902) of Yokohama City University.

### Acknowledgement

We would like to express our gratitude to Health Mock Lab project team and Nishiyodogawa Ward for providing the location for data collection in this study. We also extend our thanks to the staff of the Social Medical Corporation Aijinkai for their cooperation in this research.

### Author Contributions

NM and SN were responsible for research design and investigation, methodology, resources, data validation and visualization, and drafting the manuscript. RU and TK were in charge of game production and management. AK, MF, and HS coordinated and executed the event. SN also handled project management. TT supervised the research. HU and YO reviewed and edited the manuscript. All authors approved the final version of the manuscript.

### Approval by Institutional Review Board (IRB)

Approved by the Chibune Hospital Ethics Committee in July 2022 (No. 20230315A)
